# Safe Staffing Standards for Pharmacy Technicians in Hospital Settings

**DOI:** 10.3390/jmahp13030045

**Published:** 2025-09-04

**Authors:** Vítor Silva, João José Joaquim, Shane Desselle, Samantha Quaye, Cristiano Matos

**Affiliations:** 1 Unidade Local de Saúde de Coimbra, Praceta Prof. Mota Pinto, 3000-075 Coimbra, Portugal; vitorahsilva@gmail.com; 2 Escola Superior de Tecnologia da Saúde, Instituto Politécnico de Coimbra, 3046-854 Coimbra, Portugal; jjj@estesc.ipc.pt; 3 College of Pharmacy, Touro University California, Vallejo, CA 94592, USA; sdessell@touro.edu; 4 Pharmacy Technicians of Colour, London, UK; samantha.quaye@nhs.net; 5 European Association of Pharmacy Technicians, 1080 Brussels, Belgium

**Keywords:** pharmacy technicians, safe staff levels, hospital pharmacy

## Abstract

Pharmacy technicians (PT) are vital to the efficient and safe operation of hospital pharmacy services, fulfilling a range of technical and clinical responsibilities that directly impact patient care. However, increasing healthcare demands have underscored the importance of adequate staffing levels to sustain service quality and safeguard patient outcomes. This perspective paper explores how appropriate staffing levels for PT in hospital settings are essential and important to support safe, efficient care and a sustainable workforce. It compares evidence-informed staffing models, highlights real-world benchmarks, and proposes governance recommendations to guide policies that strengthen pharmacy services. Recommendations are made to inform clinical governance, suggesting that staffing policies, continuous training, and professional development programs are essential to supporting PT effectiveness and retention. The findings advocate for regulated staffing ratios and governance measures to foster an environment where PTs can deliver high-quality care and uphold safety standards within hospital pharmacies.

## 1. Introduction

Pharmacy technicians (PTs) play an important role in pharmacy multi-professional teams, managing medication distribution and ensuring patient safety through a variety of technical, clinical, and administrative tasks within the pharmacy sector [1]. With the increase in healthcare demands in recent years, hospital pharmacy underwent growing pressure to maintain high standards of efficiency and ensuring patient safety [2]. As the focus of healthcare shifted to patient-centered care, the PTs’ role is increasingly recognized as a major part of the medication use process [3]. In recent years, and in different countries, PTs started performing a number of tasks in clinical settings, such as medication reconciliation, immunization, and medication management, as well as helping to improve patients’ health literacy [3,4,5,6,7].

Correct staffing and workload levels form the foundation of healthcare service quality and sustainability [8,9]. These levels must be consistently ensured across the entire continuum of care, from admission to discharge, including outpatient services, to optimize patient care, enhance safety, and promote efficient use of resources [8,10,11]. When staffing at each stage of care is aligned with the complexity of the patient and the demand for service, it allows for integrated interventions, reducing the risks such as drug errors, communication failure, and care delays [12,13]. This continuity is particularly critical in transitional moments, where the likelihood of adverse events increases [12,14]. Therefore, ensuring adequate employees in the way of care is not just a question of operational efficiency, but a basic requirement for high quality, patient-centered care [13,15]. When staff are over-worked, quality of care can decline, leading to higher risks of errors, compromised patient safety, and diminished health outcomes [16,17]. Moreover, insufficient staffing contributes to increased workload, which often leads to burnout, job dissatisfaction, and higher turnover rates among healthcare professionals [13,18]. This, in turn, creates a cycle of strain on the remaining staff, further jeopardizing the stability of healthcare services [18]. As healthcare systems face growing patient populations and more complex demands, ensuring sustainable staffing levels becomes even more critical [13].

In pharmacy departments, these factors can directly impact safety and quality outcomes [16]. Insufficient staffing often leads to excessive workloads, increased number of overtime worked hours, and potentially increasing the risk of medication errors, reducing service accessibility, and affecting the job satisfaction and retention of professionals [8]. In fact, one study a number of years ago found staff shortages among the primary factors contributing to PT errors; moreover, pharmacy technicians in that same study indicated that while they “get along” with their pharmacist supervisors, those pharmacists did not seize the opportunity to further develop them after committing such an error [19]. In all likelihood, this has been further exacerbated in recent years by the COVID-19 pandemic and by stagnant wages, making it more difficult to attract pharmacy technicians into these careers let alone maintain them within the profession [20]. Yet, pharmacist leadership has been shown to go a long way in improving pharmacy technician satisfaction, mitigating turnover, and helping them as part of a future with the pharmacy organization [2,21].

The critical issues of PT staffing levels, have gained attention as determinants of both operational effectiveness and quality of care [22]. Understanding safe staffing levels for PT on whether these professionals can effectively fulfill their responsibilities while maintaining high standards of patient care is important [9,22]. Studies suggest that insufficient staffing may compromise not only operational efficiency but also patient safety, as it increases the likelihood of errors, burnout, and decreased quality of work life [23,24,25]. Moreover, the American Society of Health-System Pharmacists (ASHP) Practice Advancement Initiative 2030 highlights the importance of optimizing pharmacy technician roles through appropriate staffing and training, reinforcing their contribution to safe, patient-centered services across health systems [11]. It is essential to have guidelines that define the appropriate staffing ratios relative to the workload in all types of pharmacies, whether hospital-based or community-based [8,26]. With growing demands and complexity around providing pharmacy services, there exists a distinct shortage of cohesive regulations or evidence-based guidelines that address what constitutes effective pharmacy technician-to-workload rates [22,27]. This disparity can be experienced in different pharmacy settings (e.g., hospital pharmacies, community pharmacies, general practice) whose staffing is approached in an inconsistent way without considering patient acuity, volume of service, and complexities of task. ASHP-led initiatives along with a recent systematic review all underscore a need for a clear set of staffing parameters to protect patients, enhance workforce productivity, and develop sustainable pharmacy workforce activities in all settings of care [11,22]. Thus, any efforts at the development and implementation of staffing standards based on pharmacy practice realities should be high priorities for future health policy and workforce planning.

This perspective aims to explore the relationship between PT staffing levels and workload, analyzing current evidence, challenges, and potential models for improvement. Specifically, it seeks to examine the diverse roles and responsibilities that PTs fulfill in hospital settings, emphasizing their contributions to patient safety and service efficiency such as inpatient dispensing, sterile compounding, clinical support, and inventory management—emphasizing how appropriate staffing levels in each area contribute to patient safety and service efficiency. By assessing the impact of staffing levels and workload on the quality of care, patient safety, and job satisfaction, this research intends to provide a comprehensive understanding of the challenges posed by insufficient pharmacy technician staffing.

## 2. The Role of Pharmacy Technicians in Hospital Settings

PTs are indispensable members of the healthcare system, particularly in hospital pharmacy services, contributing to the safety and quality of pharmaceutical care [28]. In hospitals, they undertake preparing, compounding, and distributing medications, managing inventory, and ensuring the accuracy and safety of medication administration to enhance patient care [6]. In addition to dispensing and stock management, PTs often assist with handling complex medications and efficiently managing different automated dispensing technologies [6].

PTs are increasingly involved in clinical roles that enhance patient safety [22,28]. For instance, in a qualitative study, Seston et al. (2019) evaluated the implementation of a PT-supported medicines administration service designed to reduce omitted doses in hospitals [29], preventing medication errors and ensuring that patients received their medications as prescribed, which improves patient outcomes but also allows pharmacists to focus on more complex clinical tasks [29].

Another role, already performed by PTs in some countries is medication reconciliation [30,31,32]. It entails taking a medication history when the patient changes care setting, then checking, documenting, and comparing the patient’s medication records to prevent discrepancies that can lead to adverse drug events [33,34]. Recent studies have shown that PTs are effectively able to obtain medication histories and reconcile medications with comparable accuracy to other healthcare professionals such as pharmacists and nurses [35,36]. This is particularly important during hospital admissions and discharges when accurate medication information is important for continuity of care [22]. Medication reconciliation comports with other roles embraced by pharmacy, including those of pharmacy technicians, to advance public health. PTs are also involved in vaccination services [7]. While many regard these services as occurring primarily in the community setting, pharmacy technicians are also vital in efforts undertaken by hospitals and health systems to improve public health and garner the public’s support for the integration of health along of continuum of settings [37,38].

In the hospital setting, PTs can handle hazardous medications, including antineoplastic agents, ensuring that protocols for safe handling are rigorously followed [22]. This participation underscores the need for well-trained and adequately experienced professionals to manage high-risk tasks effectively, which in turn enhances the safety and reliability of healthcare services [22,28,39,40]. Such activities require strict implementation of safety practices to safeguard not only the staff, but also the patients. The United States’ National Institute for Occupational Safety and Health (NIOSH) states that working with hazardous drugs may pose extensive health risks in the absence of adequate protection [5]. Adequate staffing levels are important in these sectors to ensure safe practices and avoid rushed procedures that may compromise healthcare professionals safety and the patient quality of care [4]. Well-trained PTs can reduce or eliminate contamination and potential exposure to hazardous drugs during the preparation and administration process substantially [4]. Several countries’ guidelines advocate for specialized training and safety programs, particularly in oncology and compounding [28,41,42,43,44]. Moreover, adequate staffing with its turn provides the continuous quality improvement, such as audits, feedback process, and safe workflow [9]. PTs can also contribute to improved medication safety when teams are well-staffed and well-trained, which further enables an increase in the effectiveness of healthcare through more attention by pharmacists to complex clinical work [45]. Overall, a pharmacoeonomic evaluation has demonstrated cost-effectiveness in pharmacy operations from PT operating with a wider scope of practice [46].

## 3. The Impact of Pharmacy Technician Staffing Levels on Workflow and Service Efficiency

In recent years, the demands on pharmacy staff have intensified, driven by increased patient loads and complex medication needs [47], both for pharmacy technicians and pharmacists. As workloads expand, the need for appropriate staffing levels becomes more critical, and shortages of primary care professionals in pharmacy and elsewhere are under increased debate and scrutiny [47,48]. As healthcare systems face increasing demands due to aging populations, chronic diseases, and complex treatments, PTs have assumed expanded responsibilities [5,9,23]. According to Magrum et al. (2023), approximately 63 percent of PTs report that their workload is unmanageable, whereas data from the U.S. reveals ongoing shortages of trained staff [44,49]. Research indicates that hospital- and community-based pharmacists are willing to help increase the range of operations PTs cover in terms of efficiency [47]; they simply need to demonstrate the transformative leadership behaviors necessary in doing so [42].

The solution to workforce shortages should be multifaceted and well-structured, involving investment in targeted training, stronger regulatory frameworks, and strategies to support career retention and professional development, in addition to aforementioned leadership by supervisors of PT, who require additional training to provide that leadership [45,50,51]. Proper staffing levels enable a balanced distribution of tasks, allowing PTs to manage their responsibilities effectively [52], focus on specific duties without being forced to handle multiple roles simultaneously [53], avoid becoming overburdened, and ensure that medication is processed accurately and that potential errors are reduced [53].

On the other hand, when staffing is reduced, PTs are required to multitask, leading to increased interruptions and delays [52]. Moreover, low staffing levels force other healthcare professionals, such as pharmacists or nurses, to often pull away from clinical responsibilities to handle other tasks, with the added burden not only disrupting the patient care but also diminishing the overall quality of service [54]. When healthcare professionals, including PTs, experience staff shortages, the remaining workforce faces a disproportionate workload, often resulting in fatigue, reduced concentration, and time pressure [55]. Overburdened PTs may inadvertently overlook key steps in protocols, mislabel medications, or delay urgent dispensing activities, thereby compromising patient safety [23,24,25]. Such cases are not exclusive to pharmacy technicians: experience indicates that the lack of workforce in any profession related to healthcare (such as nurses and physicians) greatly contributes to an elevated workload on the rest of the staff and may jeopardize the level of the patients’ care and safety. Michaeli et al. (2024) refer to the overloading of physicians and nurses due to the shortages as an issue that causes fatigue and substandard care [25]. These results justify the idea that the proper staffing within every professional group is the key determinant of the overall performance and safety of healthcare services [23,24,25].

According to the Pharmacy Technician Certification Board (PTCB) 2022 National Pharmacy Technician Workforce survey, despite adequate levels of satisfaction with their careers, the profession continues to suffer from pharmacy technician shortages, where more than 60 percent of those surveyed reported a significant increase in workload in recent years [56]. The staffing shortages may be further augmented by high rates of workplace stress leading to inefficiencies and stagnation in the workflow, which can potentially compromise the quality of service delivery and the wellbeing of pharmacy technicians [56]. These challenges are also worsened by structural issues like low wages, and scarce opportunities for promotion [14,56]. The U.S. Bureau of Labor Statistics estimated that employment of pharmacy technicians will increase by 7 percent between 2020 and 2030, which is above the average growth of all occupations. Greater emphasis on task delegation by pharmacists to PTs enables pharmacists to focus their efforts on direct patient care services [47]. As such, university curriculums and healthcare systems must work jointly in competency-based training that will help them delegate properly and take on emerging roles in contemporary care paradigms [14,57].

When pharmacy technicians face high workloads due to insufficient staffing, responsibilities may be rushed or overlooked, increasing the risk of errors and impairing workflow [58]. Dabrowski & Lawrie (2021) note that such conditions contribute to bottlenecks in the medication-use process, compromising both efficiency and patient safety [4]. Magrum et al. (2023) also emphasize that at advanced workload levels, it becomes necessary for the PTs to choose speed over action, which only enhances the risk of errors, not only jeopardizing safety but actually resulting in greater amount of time spent in routine activities [44]. When it comes to emergency departments and critical care units, any delays in the availability of medications have serious implication to the patient’s outcomes even when they are very small. Consequently, adequate PT staffing ensures safety, accuracy, as well as the maintenance of efficiency and timeliness of pharmaceutical care. Furthermore, well-staffed teams are better equipped to manage inventory, track medication recalls, and respond to patient inquiries regarding drug usage, contributing to an environment where safety concerns are promptly identified and addressed [59]. Insufficient staffing can lead to delays that affect not only patient care but also operational delivery in hospitals [60]. This comparative impact is summarized in Table 1.

## 4. Safe Staffing Levels for Pharmacy Technicians in the Hospital Setting

The definition of criteria for safe staffing levels can be guided by national and international regulations; however, in most countries they are non-existent, specifically for hospital pharmacies. The World Health Organization (WHO) offers guidelines that serve as a global reference for determining staffing needs in pharmacies, with a focus on ensuring that staffing is sufficient to handle the workload and complexity of tasks [8].

The “*Workload Indicators of Staffing Need*” (WISN) manual provides a systematic approach for estimating workload and translating it into staffing requirements [8]. Its purpose is to help healthcare organizations, both hospitals and community pharmacies, to plan the number of professionals to ensure safety, quality, and availability of the services delivered. Apart from WHO, there are other guidelines which are specific from the International Pharmaceutical Federation (FIP) and ASHP, which supports the WHO model. International bodies and organizations like the FIP have developed strategic guidelines alongside quantitative models like WHO WISN, in which the role of pharmacy technicians is instrumental in the delivery of healthy and efficient pharmacy services. The 2018 Pharmacy Workforce Intelligence: Global Trends report emphasizes that the role of pharmacy technicians is more involved in different areas of healthcare, especially in hospital settings, and encourages national workforce planning strategies encompassing the role of pharmacy technicians [39]. FIP suggests coherent transfer of duties and responsibilities between pharmacists and pharmacy technicians with the focus on promoting professional development, uniform training, and regulatory acknowledgment of the pharmacy technician profession [39]. These steps will be aimed at not only proper staffing but also an optimal workforce assignment, especially as healthcare needs increase and pharmacists assume more complex clinical roles. Even though the FIP lacks a numerical staffing model, its recommendations create a normative framework through which workforce policy development can occur and inform safe staffing standards, specifically in regard to pharmacy technicians within community and hospital areas [39].

FIP stresses the issue concerning the distribution of tasks and the continuous professional development of pharmacy technicians considering the broad and expanding range of functions in the context of the expansion of roles throughout various sectors, in particular, hospitals [39]. Thus, these international organizations’ guidelines are most important for pharmacies across the globe to improve strategic staffing needs for its operations for the dynamics inherent in the demand and local conditions within the healthcare systems [8].

The main risk indicators associated with inadequate staffing include medication errors, professional burnout, and increased absenteeism [18,61]. These errors could be mitigated with appropriate funding, which would allow for more rigorous supervision and the implementation of preventive measures, such as double checking by pharmacy technicians [63].

Furthermore, Auta et al. (2015) highlighted the importance of a well-structured career plan for PT, which encourages specialization and professional development [64]. Creating a clear career structure for PTs, which involves more clinical responsibilities, improves not only talent retention but also the quality of services provided, ensuring that pharmacists can focus on their clinical roles more effectively. Yet, these also should be accompanied by a career laddering mechanism for PTs, which solidifies a future in the organization for them, imparts professional commitment, and outlines future goals for development and various organizational rewards such as upward mobility and salary increases [65].

### 4.1. Models to Determine the Optimal Staffing Levels for Pharmacy Technicians

While staffing is a critical factor in healthcare organizations, deciding the right number of PTs is an important concern in the pharmacy department. This quantity should take into account several aspects, such as the total number of beds within the hospital covered, scope and complexity of services offered, and a certain role assigned to the pharmacy team [6,22]. As an example, the departments that deal with special fields, such as oncology, pediatrics, parenteral nutrition, or sterile compounding may require dedicated and highly trained staff that can be commissioned on a full-time basis [28,44].

Several models have been found to assist managers to properly deploy staff in line with workload, demand, and difficulty. Beyond the aforementioned WHO WISN, another model is the Full-Time Equivalent (FTE) calculation model, which assesses staffing by evaluating the number of hours required to meet demand while factoring in leave and training time, which is important for continuous coverage in settings that operate 24/7 such as hospital pharmacies [66,67]. As an example, in the study by Gupta et al. (2007), which examined 110 U.S. hospitals of various sizes, they found that on average, hospitals with over 400 beds had a mean number of FTEs in the pharmacy of 43, whereas hospitals with less than 200 beds had an average of approximately 16 FTEs [66]. However, when efficiency is measured in terms of occupied bed and admission, larger hospitals proved to have been more efficient indicating that the FTE model, when calibrated to scale and complexity, can be effective at managing human resources in hospital pharmacy effectively. In support, Lee et al. (2024) established standardized staffing calculation criteria applicable to hospital clinical pharmacies in South Korea, relative to the average clinical work duration and pharmacist effective working hours, which further supports the logic of the FTE model as an approach to establishing safe staffing levels based on actual workload needs [67].

Queueing Theory Models provide another approach, analyzing workflow and wait times to optimize staff allocation. These models are particularly valuable in high-traffic hospital pharmacies, as they help minimize delays in the preparation and dispensing of medications, which can otherwise lead to longer patient waiting times for treatment administration [17]. For example, in a busy outpatient pharmacy, a queueing theory analysis showed that adding one to two extra pharmacy technicians during peak lunch hours reduced average patient wait times from 30 to 10 min [17].

The Task-Based Staffing Approach measures the work of all pharmacy technicians in hours and the time each task takes, and gives an accurate result in terms of staffing requirements [68]. This promotes the reengineering of processes and transparency. As another example, a mid-size hospital pharmacy identified that its entire medication preparation and reconciliation procedure could be mapped, and that it would require an addition of 1.5 FTE pharmacy technicians to handle measurement of volume when its clinical pharmacy services expanded.

The Staffing Model Based on Benchmarking, based on peer institutions, is used to set pharmacy technician-per-bed or pharmacy technician-per-prescription ratios to communicate with decision-makers and provide support for the staffing rates [69]. As an example, one hospital may benchmark their pharmacy technician staffing ratio with national statistics where hospitals are staffed, on average, at one pharmacy technician to every 25 beds in acute/general wards, and adjust its ratio, by higher acuity to oncology or acute care units.

FIP Strategic Workforce Guidelines present a normative structure that does not include numeric formulae but rather suggests defining technician roles and stimulating standard training, as well as making pharmacy technicians an object of national planning strategies in the field of workforce [39,70]. As an example, such guidelines can be applied in a hospital pharmacy department, to construct clear role definitions of the pharmacy technicians working in sterile compounding, handling of hazardous drugs, medication reconciliation, and designing relevant training and recognition of staff within the overall workforce plan.

These frameworks present a wide range of evidence-informed alternatives that pharmacy managers could apply to adjust staffing strategies to their own realities. Hospital pharmacies can apply a model or combination of appropriate models that address the complexity of their workload, staffing in proportion to service demand and, finally, promote safe and effective care delivery patterns. Table 2 provides a comparative overview of these evidence-informed staffing models, highlighting key advantages, limitations, and examples for practical application in hospital pharmacy contexts.

### 4.2. Recommendations for Clinical Governance Practices in Hospital Pharmacy

Based on the evidence presented, it is possible to formulate recommendations to ensure safe supply in hospital pharmacies. First, healthcare managers must implement regular internal audit systems to monitor the work efficiency and safety of hospital pharmacy tasks [8]. These audits should include a detailed analysis of workload and staffing needs to ensure staffing is appropriately sized to handle the volume of work [8] and should ensure that staffing levels reflect workload and continuity requirements, as highlighted in UK Hepatology Pharmacy Staffing Standards, which recommend a minimum 20% additional resource to cover leave and ensure sustainable service delivery [70]. To exemplify this idea, managers would be able to predict and plan any staffing changes and avoid overworking people more effectively by monitoring the number of hours of overtime worked on a regular basis or pinpointing services which might always require more employees to work on them. This is especially relevant given the example of the UK Pharmacy regulator’s (the GPhC) general approach, which highlights the risks of leaving staffing decisions solely to owners without enforceable minimum standards [71].

Furthermore, it is essential that pharmacies adopt ongoing training and professional development programs for PTs. These programs can improve the quality of care provided, and increase professional satisfaction, reducing turnover and absenteeism [60], and leading to better performance of professionals [68]. Introducing mentoring programs and creating opportunities for PTs to take on leadership roles within hospital pharmacies are seen as good practices [60,72]. Moreover, pharmacists must be mentored, themselves, to better appreciate their PT staff, to delegate effectively, and balance PT duties with the goals and missions of the pharmacy organization [73,74].

Finally, clinical governance policies should recognize the essential role of PTs in ensuring patient safety and operational efficiency. This includes ensuring that PTs receive adequate compensation and have access to working conditions that promote work–life balance [60].

### 4.3. Practical Staffing Ratios in Hospital Pharmacy

Achieving staffing ratios of PTs to patient numbers, which work in hospital settings, is a crucial factor to assess the efficiency of operations, patient safety, and quality of care. Although there are not fixed legal demands of these ratios in individual countries, there are benchmarking projects and workload assessment processes having been developed to serve as guides to staffing. This section deals with the hospital pharmacy real-life staffing ratios in the United Kingdom and the United States as well as the relevance of staffing ratios in positioning the staffing and the patient care needs.

In the UK, the NHS Benchmarking Network provides valuable insights into pharmacy technician staffing ratios across various healthcare settings. According to their findings, NHS hospitals report an average of one pharmacy technician for every 25 beds in general wards. However, this ratio tends to be lower in specialized units such as intensive care or oncology wards, where the complexity of care and medication management increases [69]. For instance, the UK hepatology pharmacy workforce guidelines recommend ratios such as 1–1.5 pharmacists per 20 beds for general hepatology patients, highlighting how clear staffing benchmarks help align workforce with patient acuity [70].

The NHS has also advocated for the implementation of workforce planning tools that allow hospitals to assess their staffing needs based on patient acuity and clinical demand. For instance, the NHS Employers’ organization emphasizes the need for flexible workforce models that can adapt to changes in service delivery requirements and patient volumes, allowing for better alignment of pharmacy technician roles within the multi-professional healthcare team [4]. The General Pharmaceutical Council’s (GPhC) current guidance places the responsibility for setting staffing levels largely on pharmacy owners, with no clear minimum ratios for pharmacy technicians, which has raised concerns about consistency in ensuring patient safety [71].

## 5. Conclusions

PTs must operate safely and efficiently, and staffing adequacy is essential for safe, efficient patient care across pharmacy services. Data shows that these smaller teams are more vulnerable to medication errors, stress, and job dissatisfaction, leading to less-than-ideal care, or poor state of professionals. On the other hand, safe and adequate staffing allows these professionals to fulfill their duties efficiently and safely, which enhances the work environment and enhances health outcomes. Organizations and professionals must engage in proper workforce planning and development and mentoring of pharmacy technicians. In addition, regulators and patient safety groups should play an active role in supporting the implementation of evidence-informed staffing standards that protect both professionals and patients.

## Figures and Tables

**Table 1:** Comparative impact of proper staffing and reduced staffing levels for pharmacy technicians.

Proper Staffing	Reduced Staffing
Enables balanced distribution of tasks, preventing overburdening of PT [22,52]	Leads to overburdened PT who must handle multiple tasks, increasing interruptions and delays [22,52]
Allows PT to focus on specific duties with accuracy, reducing the likelihood of errors [4,55]	Increases risk of errors due to multitasking, fatigue, and time constraints [17,44]
Ensures thorough safety checks on prescriptions, minimizing risks of dosage or labeling errors [44,58]	Important safety checks may be rushed or overlooked, increasing medication error risks [44,58]
Enhances workflow efficiency, supporting timely dispensing of medications [44,58]	Causes workflow inefficiencies, with delays in medication dispensing, especially in urgent situations [4,52]
Improves patient safety by allowing PT to identify and address potential safety concerns [59,61,62]	Reduces overall patient safety, as staff may lack time for essential checks and error correction [58,61]
Supports effective management of inventory and timely response to medication recalls [59]	Impacts inventory management and recall tracking, potentially delaying access to critical medications [58,59]
Reduces patient wait times for treatments and improves accuracy in drug dispensing [52,53]	Increases patient wait times and may compromise the accuracy of drug dispensing [4,52]
Facilitates efficient use of technology, allowing pharmacists to focus on complex clinical tasks [5,53]	Limits the ability of staff to effectively operate technology, impacting workflow and clinical focus [5,18,53]
Associated with lower burnout rates and improved morale among PT and other staff [18,44]	Leads to greater stress and risk of burnout, decreasing job satisfaction and patient care quality [18,25].

**Table 2:** Comparative models to determine the optimal staffing levels for pharmacy technicians.

Staffing Model	Evidence/Source	Advantages for Hospital Pharmacy Staffing	Limitations for Hospital Pharmacy Staffing
Workload Indicators of Staffing Need (WISN) [8].	World Health Organization; adapted to healthcare facilities including pharmacy departments	- Tailors staffing to actual workload data, helping managers align pharmacy technician shifts with medication preparation, dispensing, and inventory demands. - Reduces PT burnout by matching staffing levels to peak demand times, improving job satisfaction and patient safety.	- Requires ongoing, accurate data on task frequency and duration, which may be difficult to collect consistently. - Limited flexibility for sudden changes in demand or emergency situations.
Full-Time Equivalent (FTE) Calculation [66,67].	Commonly used in hospital settings for 24/7 operations, including pharmacy departments	- Ensures comprehensive coverage by accounting for PT leave, training, and shift needs. - Useful in 24/7 hospital settings where continuous service is critical for safety.	- Based on historical workload data, which may not reflect sudden increases in demand. - May lack flexibility to respond to daily workload variability or emergencies.
Task-Based Staffing Approach [68]	Davidson et al. (2021) [68]	- Maps all PT technician tasks and time needed, providing precise staffing estimates. - Supports process reengineering and workload transparency.	- Requires comprehensive task analysis and systematic data collection. - May not adapt well to sudden fluctuations in workload.
Benchmarking-Based Staffing Model [69]	NHS reports	- Uses peer institution data to establish technician-per-bed or technician-per-prescription ratios. - Facilitates comparison and communication with decision-makers.	May not reflect local complexity or workload specifics. Requires access to standardized, comparable external benchmarks.
Queueing Theory Models [17]	Applied in high-traffic hospital pharmacies to manage PT deployment and reduce patient wait times	- Optimizes staffing during high-demand periods, helping technicians efficiently manage dispensing and patient inquiries during peak hours. - Reduces wait times and workload by analyzing patterns, thus improving overall service quality and PT technician workflow.	- Requires regular data analysis and adjustments, which may be challenging in fast-paced hospital settings. - More effective in high-traffic pharmacies and less applicable in smaller or lower-traffic units.
FIP Strategic Workforce Guidelines [39]	Pharmacy Workforce Intelligence: Global Trends Report 2018	- Provides a normative framework for defining technician roles, promoting standardized training, task delegation, and inclusion in national workforce planning. - Supports quality, safety, and adaptability in PT staffing, particularly in hospital settings.	Does not offer numerical formulas or staffing ratios. Implementation depends on national policy uptake and regulatory context.

FIP—International Pharmaceutical Federation; FTE—Full-Time Equivalent; NHS—National Health Service (UK); PT—pharmacy technician; WHO—World Health Organization; WISN—Workload Indicators of Staffing Need.

## Data Availability

Requests to access the datasets should be directed to the corresponding author and will be granted upon reasonable request.

## Abbreviations

The following abbreviations are used in this manuscript:

ASHP	American Society of Health-System Pharmacists
FIP	International Pharmaceutical Federation
FTE	Full-Time Equivalent
GPhC	General Pharmaceutical Council
NHS	National Health Service
PT	Pharmacy Technician(s)
WHO	World Health Organization
WISN	Workload Indicators of Staffing Need
